# Bacterial vaginosis: drivers of recurrence and challenges and opportunities in partner treatment

**DOI:** 10.1186/s12916-021-02077-3

**Published:** 2021-09-02

**Authors:** Lenka A. Vodstrcil, Christina A. Muzny, Erica L. Plummer, Jack D. Sobel, Catriona S. Bradshaw

**Affiliations:** 1 Central Clinical School – Melbourne Sexual Health Centre, Monash University, 580 Swanston St, Carlton, Victoria 3053 Australia; 2 Melbourne Sexual Health Centre, Alfred Health, Carlton, Victoria Australia; 3 Melbourne School of Population and Global Health, The University of Melbourne, Parkville, Victoria Australia; 4 Division of Infectious Diseases, University of Alabama at Birmingham, Birmingham, AL USA; 5 Division of Infectious Diseases, Wayne State University, Detroit, MI USA

**Keywords:** Bacterial vaginosis, Male partners, Female partners, Treatment, Vaginal microbiota, Urethral microbiota, Penile microbiota, Metronidazole, Clindamycin, Sexually transmitted infection

## Abstract

Bacterial vaginosis (BV) is the most common vaginal dysbiosis to affect women globally, yet an unacceptably high proportion of women experience BV recurrence within 6 months of recommended antibiotic therapy. The low rate of sustained cure highlights our limited understanding of the pathogenesis of BV recurrence, which has been attributed to possible persistence and re-emergence of BV-associated bacteria (BVAB) or a BV-associated biofilm following antimicrobials and/or reinfection occurring from sexual partners.

There is a robust body of evidence to support the exchange of bacteria between partners during sexual activity, and while the hypothesis that women treated for BV are subsequently reinfected with BVAB following sex with an untreated sexual partner is not new, failure of past partner treatment trials has eroded confidence in this concept. If reinfection is a key driver of recurrence, current antimicrobial regimens directed to women alone are unlikely to achieve a high level of sustained cure, and the approach of partner treatment to reduce reinfection is justified. In this manuscript, we present the molecular and epidemiological evidence that underlies the hypothesis that BV is sexually transmitted, and summarise why research that continues to consider sexual partnerships is necessary. We also outline the significant barriers and challenges that we have identified while undertaking partner treatment studies, and we discuss the factors that impact on our ability to determine their effectiveness.

Ultimately, the pathogenesis of BV recurrence is likely to be multifaceted and not attributable to a single mechanism in all women. If we are to achieve sustained cure for women, it is likely that combined and individualised approaches to eradicate BVAB, support an optimal vaginal microbiome, and prevent reinfection from partners will be required.

## Background

Bacterial vaginosis (BV) is the most prevalent vaginal condition, affecting 30% of women globally [1]. BV is associated with an increased risk of a broad range of gynaecological and obstetric sequelae including preterm delivery, spontaneous abortion, early pregnancy loss in IVF, and HIV/STI acquisition and transmission [2–7]. Although up to half of BV-affected women do not experience symptoms [8, 9], for those that do, it is the symptoms themselves, including malodour and vaginal discharge, that cause significant distress to women and impact on their quality of life and relationships [10]. Current evidence indicates that BV is a polymicrobial syndrome characterised by a shift in the composition of the vaginal microbiota from ‘optimal’ to ‘non-optimal’ [11–14]. This non-optimal microbiological state involves a reduction in protective lactobacilli, and an increase in bacterial diversity and facultative and strict anaerobes, including *Gardnerella* spp., *Atopobium vaginae*, *Prevotella* spp., and others, referred to as BV-associated bacteria (BVAB) [11, 15]. While the exact pathogen/s responsible for BV are still debated, a recent conceptual model hypothesised that virulent strains of *Gardnerella*, as well as *Prevotella bivia* and *A. vaginae*, play a central role [16].

**Fig. 1:**
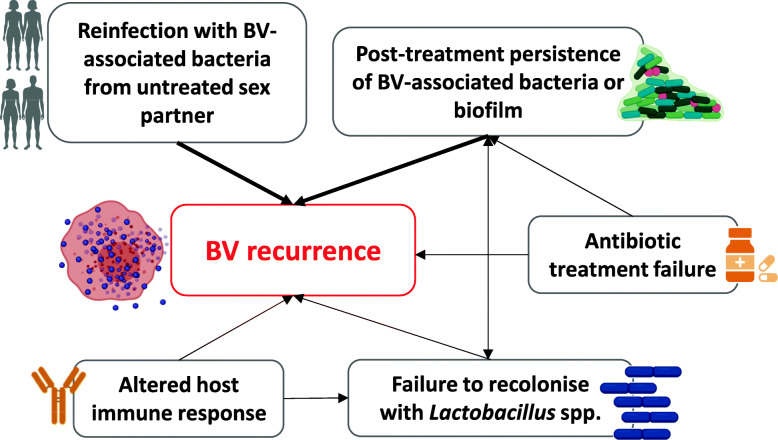
Factors hypothesised to drive BV recurrence. Thicker arrows indicate main hypothesised drivers of BV recurrence

Recommended first-line antimicrobial treatments, metronidazole or clindamycin [17–20], provide broad anaerobic coverage and are administered orally or intravaginally. These regimens have similar efficacy and cure ~70–85% of women with BV within 1 month [21, 22]; however, more than 50% experience recurrence of symptoms and/BV on microscopy within 6 months [23, 24]. The low rate of sustained cure not only highlights our incomplete understanding of the pathogenesis of BV recurrence, but also compounds women’s distress and frustration, and leads to repeated presentation to health services or adoption of unproven home-remedies [10]. Factors including persistence of a BV-associated biofilm, failure to recolonise the vagina with lactobacilli, reinfection from an untreated partner, and host genetic and/or immune factors may all play a role in recurrence (Fig. 1) [25–29]. Frustratingly for both clinicians and patients, the contribution of reinfection and vaginal relapse cannot be separated, as the clinical presentation of both mechanisms of recurrence is identical. Point-of-care laboratory tests cannot resolve this issue yet, and molecular methods, including next generation sequencing, are unable to identify a unique or specific microbial signature to separate the two routes of recurrence [11, 15, 30]. While persistence or resistance of BVAB or BV biofilm following antibiotic therapy is likely to be the dominant mechanism among some women, there is a robust body of evidence to support the exchange of both optimal and detrimental bacteria between partners during sexual activity [31–35]. These data suggest that reinfection of women with pathogenic BVAB may be a key driver of recurrence following treatment [16, 26, 27, 36, 37]. If this is the case, current antimicrobial regimens directed solely to women are unlikely to achieve a high level of sustained cure.

Clinicians and researchers agree that there is an urgent need to develop more effective treatments to improve BV cure, and ultimately reduce adverse health outcomes [38]. In this review, we will discuss the disconnect between the epidemiological and microbiological evidence supporting the contribution of reinfection from an untreated sexual partner to the BV-syndrome, and the outcomes of prior trials that have evaluated partner treatment to improve BV cure. We will outline the opportunities that sexual partner treatment may afford to women with BV and discuss the challenges faced when conducting partner treatment trials.

## Main text

### Evidence to support sexual ‘transmission’ of the BV syndrome

There is long standing evidence to support the contribution of sexual transmission to the pathogenesis of BV. In the 1950s, Gardner and Dukes first characterised BV and hypothesised that “…husbands must be treated simultaneously if recurrences by reinfection are to be prevented” [39]. When vaginal discharge was transferred from women with BV to those without, 11/15 women developed clinical signs and microbiological features of BV [39], indicating the BV syndrome was infectious. Initially, *Gardnerella vaginalis* was isolated from women with BV and hypothesised to be a new STI responsible for the syndrome; however, when 13 ‘healthy’ women were inoculated with pure cultures of *G. vaginalis* (incubated for 24h prior to inoculation and in the stationary phase of growth), only one developed BV [39]. In a subsequent study, which also used fresh cultures of *G. vaginalis* but incubated them for 12h prior to inoculation and in the late logarithmic phase of growth, 5/9 women developed the BV syndrome [40]. This finding suggests that phase of growth and environmental signals may control expression of bacterial virulence factors which confer a greater pathogenic potential to *G. vaginalis*, a finding supported by more recent evidence [41]. In support of the initial hypothesis that *G. vaginalis* is the etiologic agent responsible for BV, it was isolated from the urethra of 91/101 husbands of women with BV, but only 1/38 male medical students [42]. Successive studies also isolated *G. vaginalis* from the urethra of male partners of women with BV [43], and from a larger population of heterosexual men [44]. The high rates of BV recurrence post-treatment and evidence of male carriage of *G. vaginalis* led to six male-partner treatment trials of women with BV in the 1980s and 1990s (Table 1) [45–50]. Only one trial found a statistically significant reduction in BV recurrence [47], and the failure of the other trials was then interpreted as evidence against sexual transmission; thus, partner treatment is not currently recommended in guidelines [18, 54]. Lessons learned from these past trials and their limitations [53–55] are discussed later in this review.

**Table 1:** Updated summary of prior male partner treatment trials aimed at reducing recurrent BV

First author (year)	Location	Sample size^a^	Treatment of women	Treatment arm for men	Comparator arm (men)	Primary outcome	Findings
Swedberg (1985) [45]	Casper, Wyoming, USA	64	Oral MTZ 2g single dose OR	Oral MTZ 2g single dose OR	Standard of care	Culture negative for *G. vaginalis* and improved vaginal symptoms at 21d	68% *v* 64% RR=1.06; 95%CI 0.74–1.52
			Oral MTZ 500mg *bid* 7d	Oral MTZ 500mg *bid* 7d			
Vejtorp (1988) [46]	Denmark	106	Oral MTZ 2g on d1 and 3	Oral MTZ 2g on d1 and 3	Placebo	Clinically diagnosed BV at 5w	25% *v* 29% RR=0.85; 95%CI 0.45–1.61
						*G. vaginalis* at 5w	26% *v* 40% RR=0.64; 95%CI 0.37–1.12
						Symptom improvement or cure at 5w	26% *v* 40% RR=0.64; 95%CI 0.83–1.29
Mengel (1989) [47]	Seattle, Washington, USA	98	Oral MTZ 2g single dose OR	Oral MTZ 2g single dose OR	Placebo	Symptoms and clinical cure of BV at 2, 5, and 8w; BV on vaginal Gram stain at 2 and 5w	No point estimates reported^b^
			Oral MTZ 500mg *bid* 7d	Oral MTZ 500mg *bid* 7d			
Moi (1989) [48]	Denmark, Finland, Norway, Sweden	190	Oral MTZ 2g on d1 and 3	Oral MTZ 2g on d1 and 3	Placebo	Relapse of clinically diagnosed BV at 12w	21% *v* 16% RR=1.33; 95%CI 0.73–2.44
Vutyavanich (1993) [49]	Thailand	133	Oral TIN 2g single dose	Oral TIN 2g single dose	Placebo	Clinical cure of BV at 4w	72% *v* 63% RR=1.13; 95%CI 0.95–1.35
Colli (1997) [50]	Italy	139	2% clindamycin intravaginal cream 7 nights	Oral clindamycin 150mg *qid*	Placebo	Clinically diagnosed BV recurrence at 12w	31.9% *v* 30.0% RR=1.06; 95%CI 0.65–1.75
Schwebke (2021) [51]	Multisite, USA	214	Oral MTZ 500mg *bid* 7d	Oral MTZ 500mg *bid* 7d	Placebo	BV cure defined as 0–2 Amsel criteria and Nugent score 0–6 at 16w	81% *v* 80% *Fisher’s exact test p>0.999*
Vodstrcil^*c*^ *in progress* [52]	Multisite, Australia	*Aims to recruit 342*	Oral MTZ 500mg *bid* 7d^d^	Oral MTZ 500mg *bid* and topical clindamycin *bid* 7d	Standard of care	BV recurrence defined as 3–4 Amsel criteria and Nugent score 4-10 at 12w	*in progress*

Adapted from Mehta [53]; “standard of care” indicates female-only treatment; *MTZ*, metronidazole; *TIN*, tinidazole; *d*, days; *w*, weeks; *m*, months

^a^Sample size reflects those included in the analysis

^b^At 2 and 5w, women who had male sexual partners who received treatment showed less BV on vaginal Gram stain; these women also had fewer vaginal symptoms at 8 weeks

^c^Trial is currently recruiting (ACTRN12618000219280)

^d^Women can receive topical clindamycin 7 nights if MTZ is contraindicated

What is clear now is that there is a substantial body of epidemiological and microbiological data to support the role of sexual transmission in the BV syndrome. As is the case for established STIs, prevalent BV is associated with earlier age of sexual debut [56–58] and is uncommon in women reporting no sexual activity [58, 59]. In a 12-month cohort study, young women engaging in penile-vaginal sex were more likely to have a vaginal microbiota dominated by *Gardnerella* spp. than women reporting no sex [60], and in a cross-sectional study of 17- and 18-year-old women, the presence and abundance of *G. vaginalis*, *A. vaginae*, and *P. bivia* was associated with history of penile-vaginal sex [61]. Furthermore, a meta-analysis showed a positive association between increased numbers of male sexual partners or a recent change of male partner and risk of BV (relative risk [RR]=1.6, 95%CI 1.5–1.8). Conversely, consistent condom use was associated with a decreased risk of BV (RR=0.8, 95%CI 0.8–0.9) [62]. The estimated protective effect of condom use for BV is similar in magnitude to the association reported between condom use and chlamydial and gonococcal infections (adjusted odds ratio [aOR]=0.82, 95%CI 0.66–1.01) [63]. Furthermore, in a systematic review of risk factors for BV among women who have sex with women (WSW), BV was associated with increased number of lifetime female sexual partners, recent female partners, and sex with a partner with a concurrent BV diagnosis [64].

### The relationship between BV acquisition and sexual practices

Incident BV has also been strongly associated with sexual practices and women reporting a new sexual partner are more likely to acquire BV [36, 65, 66]. Among women with male partners, penile-vaginal sex, inconsistent condom use, and semen exposure have been associated with BV acquisition/incidence [67, 68]. However, BV transmission can occur in the absence of semen [69], and women engaging in sex with women also acquire BV. Studies of WSW have confirmed the relationship between BV acquisition and a number of sexual practices with women including sex with a new partner, and oral, digital-vaginal, and digital-anal sex [36, 64, 66]. In one 2-year cohort study, WSW who reported a new sexual partner had a more diverse and unstable vaginal microbiota, characterised by *Gardnerella* spp. or other BVAB, and specific clades of *Gardnerella* were associated with different sexual practices [37, 70]. However, the timing between sampling and BV acquisition could not be accurately determined, as sampling only occurred at 3-month intervals. To further pinpoint the incubation period between exposure to BVAB and onset of BV, WSW providing daily vaginal specimens were followed for up to 90 days [71]. Among this cohort, an increase in the relative abundance of *Gardnerella* spp., *P. bivia*, and *A. vaginae* as well as the onset of the BV syndrome occurred within approximately 4 days of sex, an incubation period analogous to that of other bacterial STIs (i.e. chlamydia, gonorrhoea) [71, 72].

Female couples provide additional proof of sexual exchange and sharing of the vaginal microbiota. Concordance of Nugent scores (the microbiological scoring method for BV) is high in women in monogamous relationships [73–75], and couples who both have BV are also concordant for specific BVAB [74]. Meanwhile, female couples without BV share lactobacillus strains associated with optimal vaginal microbiota, and 77% of monogamous couples have identical lactobacilli strain-fingerprints [76]. Female monogamous couples who were BV-negative at enrolment did not develop BV over 24 months [36], reflecting a shared stable optimal vaginal microbiome. Only after sex with a new partner outside of the relationship did women enrolling as a couple acquire BV. However, further research is needed into how specific bacterial strains may be transmitted and contribute to BV acquisition, and/or if there are any host factors that may render someone more susceptible to acquisition when exposed to a certain strain/s.

### The relationship between recurrent BV and sexual practices

While new sexual partnerships drive BV acquisition, the risk of BV recurrence following re-exposure to the same sexual partner after treatment increases 3-fold [26, 77]. This effect size was not reduced after adjustment for frequency of sex or condom use, which suggests that sexual exposure results in the exchange of organisms, rather than ‘enhancing’ or ‘re-activating’ existing BVAB. Subsequent microbial sequencing found women who resumed sex with their ongoing partner following antibiotic treatment were more likely to have a vaginal microbiota colonised with BVAB than women without an ongoing partner [30]. Inconsistent condom use is another risk factor for BV recurrence [26, 77–79]. However, the effectiveness of condom use in preventing alterations to the composition of the vaginal microbiota needs further examination, with one study showing that women with inconsistent condom use had a similar relative abundance of BVAB to women reporting consistent use [30]. Further research is still needed to understand the key microbial drivers of recurrent BV, especially in the context of an ongoing partner that may be harbouring, and therefore reintroducing, specific BVAB.

### BVAB colonise the urethra and penile skin

Early studies isolated *G. vaginalis* from the urethra of men [39, 42–44] and concordant *G. vaginalis* biotypes from the genitals of 11/12 heterosexual couples [80]. Modern sequencing methods have provided extensive microbiological evidence of male carriage of BVAB at the penile skin and urethral sites [81]. Further, BVAB have also been shown to be more commonly detected in the sub-preputial region and distal urethra of male partners of women with BV than without [33, 82], and carriage of specific BVAB has been shown to be concordant between monogamous couples, with penile site-specific differences [31, 33–35]. In a pilot partner treatment trial, which treated male partners of women with BV with concurrent antibiotic treatment, several BVAB and *Lactobacillus* spp. were correlated between sexual partners, with a higher number of taxa correlated between the vaginal and cutaneous penile sites compared to the vaginal and urethral sites [83]. Interestingly, in the 12 weeks following treatment in couples where the female experienced BV recurrence, both *Prevotella timonensis* and *Sneathia amnii* were strongly positively correlated between the vagina and cutaneous penile site, whereas *Lactobacillus iners* was negatively correlated between the two sites. Conversely, among couples where women were cured, *Lactobacillus* spp. were positively correlated. These findings provide evidence that BVAB colonise men and indicate that taxa shared between a couple can differ depending on BV status. Fluorescence in situ hybridization studies have identified a *Gardnerella* biofilm in the urine of male partners of women with BV [84] and a polymicrobial BV biofilm in semen samples of male partners of women with BV [85]. Semen has been implicated in the BV syndrome [68], and the semen microbiota has been shown to alter the composition of the vaginal microbiota [32]. This effect has been hypothesised to be mediated by post-coital elevations in vaginal pH; however, the increase in vaginal pH is transient; and it is more plausible that semen facilitates BV transmission by increasing the inoculum of BVAB. Whether there is an upper genital tract source for these BVAB in men or whether semen simply provides a vehicle to transfer urethral BVAB is not known.

The contribution of the cutaneous penile microbiota to BV acquisition in women was evident in a large circumcision trial in sub-Saharan Africa that had a primary aim of reducing HIV transmission. Circumcision was associated with a 40% reduced risk of a female partner acquiring BV, and female partners of circumcised males carried fewer BVAB [86–88]. Furthermore, the total bacterial load and diversity of the coronal sulcus decreased 1 year after circumcision [82], with a significant reduction in the prevalence of BVAB and corresponding increase in non-BVAB. These studies suggest that among uncircumcised men, the coronal sulcus harbours higher loads of BVAB compared to circumcised men and points to the sub-preputial space as a key site for sexual exchange of BVAB. However, circumcision alone was unable to prevent BV acquisition in all women [86]. A recent prospective study following heterosexual couples demonstrated that meatal microbiota accurately predicted BV acquisition in female partners, and with more consistency than the cutaneous penile microbiota [89]. The taxa in the meatus that were considered most predictive of acquisition included *Gardnerella, Parvimonas*, *Dialister*, *Sneathia sanguinegens*, *L. iners*, and *L. crispatus* [89]. Interestingly, the composition at either penile site was able to accurately predict BV incidence at a higher rate than circumcision status [89]. Data from Plummer et al. highlight that the two penile sites harbour BVAB with differing tropism, and both may contribute to BV pathogenesis. Specifically, *Gardnerella* and *Sneathia* were abundant and prevalent at the urethral site, whereas *Prevotella* spp., *Finegoldia*, *Peptoniphilus*, and *Anaerococcus* were abundant and prevalent at the penile-skin site ([83] and *personal communication*).

Collectively, these data illustrate male carriage of BVAB and highlight how male partners and specific anatomical sites may contribute to BV acquisition and recurrence. While it is possible that some BVAB may comprise a ‘normal’ or ‘healthy’ penile skin and/or urethral microbiota, a comprehensive understanding of the male genital microbiota is needed to develop effective targeted interventions to determine if eradication or suppression of male carriage will prevent reinfection and acquisition of BV in women. Given the compelling epidemiological and microbiological evidence, it is logical to re-visit partner treatment to reduce BVAB carriage and improve long-term BV cure, taking into consideration the challenges faced in conducting these trials.

### What we can learn from past male-partner treatment trials

More than 60 years ago, researchers and clinicians held the view that the syndrome of BV was sexually transmitted, or at least, associated with sex. The findings from the six male-partner treatment trials conducted in the 1980s and 1990s have been extensively reviewed [53–55], but we briefly summarise the findings (Table 1), with the aim of understanding the outcomes.

One of the challenges when assessing the effectiveness of partner treatment is that the trials utilised very different combinations of antibiotic treatments for differing durations (Table 1), and there was no published data to inform what treatments may be optimal for men. These trials had major trial-related limitations including small sample sizes, absence of power calculations, inconsistent and non-standard methods to diagnose BV in women, use of single-dose and non-standard treatments for women (which we now know are suboptimal), lack of data on treatment adherence, and high attrition [53, 55]. Another key difference between the trials was that the clinical endpoints were measured anywhere from 7 to 10 days to 12 weeks post-randomisation (Table 1). The timing of the endpoint may be critical in determining if an intervention is efficacious. Among couples counselled to abstain from sex, BV endpoints measured immediately post-treatment are more likely to reflect the efficacy of female-only treatment rather than the effect of treating the male on BV. In contrast, endpoints measured after a longer period of time post-treatment may be undermined by the introduction of untreated concurrent partners to the relationship. Overall, these limitations render the findings of these early trials inconclusive and cannot be used as evidence that BV is not sexually transmitted or that treating male partners will never be effective.

Since these two systematic reviews were published, we have the results from a recently conducted RCT [51]. Schwebke et al. treated women with recurrent BV with the current first-line regimen of 7-day oral metronidazole 500mg *bid* and their male sexual partners were randomised to concurrent treatment with the same regimen or an oral placebo (Table 1). This trial was the most thoroughly designed and addressed the limitations of prior trials, with sufficient sample size and clear power calculations, appropriate randomisation methods, blinding, and inclusion of data on antibiotic adherence. However, the trial was stopped early due to futility, as the primary-intention-to-treat analysis showed partner treatment did not improve cure by 16 weeks’ post-randomisation. Even though these findings appear to be yet another notch against male-partner treatment, there are important considerations to note. The overall recurrence rate was 80% in women in both groups, which is higher than prior studies [23, 24], and may suggest the study population had underlying risk factors that placed them at high risk of recurrence, or persistent BV biofilm that is refractory to oral metronidazole. Further, post hoc analyses revealed that among couples where the man was 100% adherent to metronidazole treatment, the female partners were significantly less likely to recur (73% *vs.* 97% recurrence in women whose partners were 100% *vs.* <100% adherent, respectively, *p*<0.001). These findings suggest a modest effect of oral treatment in highly adherent males, but it is important to consider that oral therapy alone may also not sufficiently impact on cutaneous/sub-preputial carriage of BVAB.

### Moving forward: the challenges and opportunities of partner treatment

Clearly, there are considerable challenges in undertaking partner treatment trials (Table 2). For these trials to work, it is imperative that they attempt to enrol ‘closed-couple units’ to ensure the efficacy of the intervention is not eroded by the introduction of untreated concurrent partners prior to endpoint. Of course, this is difficult, especially as the bulk of partner treatment studies recruit women from STI clinics, where concurrent partnerships are more common, and attendees are more likely to engage in higher risk sexual practices than the general population [90, 91]. Enrolling community-based cohorts and the use of a ‘couples’ verification tool’ [92] may help to reduce the risk of enrolling a couple with external partners; however, external partnerships will not always be disclosed, with sexual behaviour data prone to bias and even more so when both individuals in a partnership are enrolled [93–96].

**Table 2:** Factors influencing the outcome of partner treatment trials

Enrolment hesitancy or resistance	
Timely recruitment of partner	
Study medication non-adherence	
Barrier contraception use, especially during treatment	
Concurrent sexual partnerships	
Loss to follow up, particularly if treatment is effective	
Selection bias of females with highly recurrent BV (potential antimicrobial resistance, persistent tenacious biofilm)	

### Partner treatment trials targeting male partners

There are an additional set of challenges when recruiting couples to male-partner treatment trials. Women are often reluctant to disclose their symptoms to men [97] and may require educational material and support to increase their confidence in discussing their BV and the associated morbidities with their partner. Motivating male partners to participate is also made more difficult by the fact that most men do not experience symptoms themselves. This was highlighted by men participating in partner treatment trials, who reported BV to have little impact on themselves beyond their concerns for their partner’s health, self-esteem, and confidence [97]. Interestingly, some men identified that the diagnosis of BV in their female partner led to improved communication with the partnership about sex and sexual health,and that accepting partner treatment showed they were committed to the relationship and could be a supportive partner [97]. Further research to identify the factors that may motivate male participants to participate in BV treatment trials is required.

Fundamentally, we also still do not know what the most appropriate and effective antibiotic agents are to use in male partners. Nearly all the RCTs to evaluate partner treatment used oral metronidazole (Table 1). Metronidazole, a nitroimidazole antibiotic with broad activity against anaerobic bacteria [98, 99], achieves 1-month cure rates of >80% among women [21, 22]. Less than 1% of anaerobic bacteria isolated from women pre-treated with 5-day intravaginal metronidazole demonstrated resistance to metronidazole in vitro [100]. However, specific *Gardnerella* clades and other potentially pathogenic organisms including *A. vaginae* have been documented to show intrinsic resistance to metronidazole [101–107], and a BV-associated biofilm has also been shown to be resistant to metronidazole treatment [25, 108]. Several other non-cultivatable organisms may also have resistance genes responsible for treatment failure [109]. Novel mechanisms by which metronidazole is ineffective have also been proposed; *L. iners* was recently demonstrated to sequester metronidazole in vitro, resulting in a lower efficacy of metronidazole against *Gardnerella* [110].

Many of the previous partner trials opted for stat 2g doses of oral metronidazole to improve adherence, and we know adherence is essential to the success of any intervention; however, the short duration of activity of this single dose may have had limited impact on the male genital microbiota [51]. Newer single-dose nitroimidazoles approved for the treatment of BV, such as 2g oral secnidazole [111], have similar efficacy to metronidazole but a longer half-life and thus may be more suited to partner treatment [112]. However, secnidazole and tinidazole have similar resistance patterns to metronidazole [113] and other drug classes or combinations of antimicrobials may be required to cover candidate BV pathogens. Clindamycin, which is commonly used in women with a contraindication to nitroimidazoles but also used first-line, is a lincosamide antibiotic with broad spectrum activity against Gram-positive cocci as well as anaerobic Gram-positive and Gram-negative bacteria. Clindamycin has been shown to have greater efficacy than the nitroimidazoles against *A. vaginae*, *Gardnerella* spp., and *Mobiluncus* spp. [113], and planktonic clinical isolates of *Gardnerella* exhibit a relatively higher susceptibility rate and lower resistance rate to clindamycin compared to metronidazole, suggesting clindamycin may be more optimal for reducing the abundance of *Gardnerella* [114]. While the effectiveness of metronidazole and clindamycin in clearing BVAB from women is still debated [115], data is needed to show if the effectiveness of these agents on BVAB in men. Newer biofilm-disrupting agents, for example dequalinium chloride [116] and TOL-463 [117], may be effective at disrupting BV biofilms in women and may also have a role in men, but this is yet to be determined. A registered RCT (NCT03412071 [118]) that aims to establish the effect of antimicrobial agents (oral tinidazole 2g for 2d, penile topical metronidazole, topical clindamycin, or topical hydrogen peroxide) on the penile microbiota may provide some insight into the effect of different antimicrobials directed to men.

It is plausible that both penile skin and urethral carriage of BVAB needs to be targeted if we are to achieve sufficient clearance of BVAB from the male genitalia and impact on reinfection in women. Two pilot partner treatment trials have targeted BVAB in men for 7 days at the distal urethra with 400mg metronidazole *bid*, and at the sub-preputial space and coronal sulcus with topical 2% clindamycin cream applied *bid* [34, 83]. Treatment had an immediate effect on the composition of the penile-skin and urethral microbiota, with a decrease in the prevalence and abundance of BVAB; however, this change was not sustained at 1–3 months [34, 83]. Despite re-emergence of BVAB in men, the female partners of treated males experienced a significant and sustained decrease in the prevalence and abundance of BVAB to endpoint [34, 83]. Although not powered to detect the effect of partner treatment, the pooled BV recurrence rate among the 50 couples undergoing treatment across both trials was only 8% at 1 month, and only 17% among the 29 couples followed to 3 months. Both are lower recurrence rates than would normally be expected in sexually active couples where a history of BV was common, as in other trials where recurrence among couples ranged from 40 to 80% [26, 51, 77]. These data raise important questions: how effective does concurrent partner treatment need to be at reducing BVAB in men, and for what duration do the organisms need to be suppressed, to elicit a positive and sustained result in the female? Effective concurrent male-partner treatment may only need to disrupt the continued cycle of exposure to BVAB for a short period of time to provide women with an opportunity to recover post-treatment to a *Lactobacillus*-dominated state, capable of withstanding re-exposure to BVAB from their partner in the future.

The dual-therapy concurrent partner treatment approach is subject to an ongoing RCT (ACTRN12618000219280 [52];). A pragmatic open-label trial design is being used, with female-only treatment as the control group (i.e. current standard-of-care) compared to concurrent-couple treatment. In addition to the primary analysis, which will assess how this approach impacts cure rates, the specimens collected in this and the Schwebke RCT [51, 52] will inform our understanding of the short- and long-term impact of these antimicrobials on BVAB in both women and men, as well as the benefit of adherence to treatment.

### Partner treatment trials targeting female partners

Despite BV being common among WSW, no partner treatment trials enrolling female couples have been conducted. As mentioned above, the majority (74–95%) of female couples have been shown to be concordant for the presence or absence of BV [36, 74, 75]. If a female index case is enrolled in a partner treatment trial, it is highly likely that her female partner will be concordant for the presence of BV. Enrolling female couples would therefore provide an opportunity to determine the effectiveness of partner treatment in both members of the partnership. However, female-partner treatment brings a unique set of additional challenges. Enrolling BV-concordant couples in a blinded randomised partner treatment trial would mean that only half of the female partners would be treated for BV at enrolment, as the rest would be randomised to placebo. The concept of a placebo arm for women may not be accepted by participants or clinicians. Apprehension surrounding the ethics of non-treatment could be mitigated by considering firstly, that it has not been shown that treating BV prevents adverse outcomes, only symptoms [18], and if one of the partners has asymptomatic-BV at enrolment, treatment would not currently be recommended [18, 119]. FDA guidance around BV treatment trials also states that a placebo can be used as a comparator for the control arm [120], and recent BV treatment trials have done so [111, 121]. Even if partner-treatment was accepted, in order to measure clinically meaningful cure, follow-up to 3–4 months is required, and it is quite likely that concerns around possible placebo therapy would lead to treatment seeking behaviour prior to endpoint. To address this, a rescue arm may need to be incorporated for partners who develop vaginal symptoms at any time during the trial so they can request evaluation and treatment of symptomatic BV and/or other vaginal infections, as is included in FDA guidance [120].

## Conclusions

It is clear that current therapies that are solely directed to women have failed to achieve a high rate of sustained cure. By continuing to ignore the potential risk of sexual transmission and reinfection when evaluating the efficacy of treatments, the effectiveness of these strategies is likely to be consistently underestimated. When managing a patient with BV, incorporating the regular partner who may be a source of reinfection provides an opportunity to make an impact on treatment outcomes. We need to continue to assess how antimicrobials change the genital microbiomes of both women with BV and their partner to ensure the most effective regimens are utilised. Furthermore, in order to overcome the short-comings of past partner treatment RCTs, we need innovative ways to engage women with BV and their partners to ensure recruitment of monogamous couples and promote adherence to treatment. Continued research into the pathogenicity of bacteria that are shared between couples may assist us in developing new treatment strategies, as well as providing evidence to support partner treatment as an adjunctive therapy to improve health outcomes for women.

Ultimately however, the pathogenesis of BV recurrence is complex, with reinfection likely to be important, but not the sole driver, of recurrence (Fig. 1). In some women, vaginal relapse (i.e. the re-emergence or persistence of BVAB) may be the dominant mechanism. There has been considerable focus recently on new therapeutic approaches aimed at biofilm eradication [116, 117], thereby removing the protected sanctuary containing bacterial pathogens and potentially eliminating a mechanism of BVAB persistence [122, 123], and some are included as alternative treatments in guidelines [19]. Similarly, evidence of antimicrobial resistance is reported; however, its contribution to recurrence is unclear and new antimicrobial drug classes have not been forthcoming [115]. The development of new molecular assays, which diagnose BV based on the specific pathogenic bacterial strains or the presence of anti-microbial resistance, may ultimately result in more precise and targeted therapies to improve cure. Given the failure of therapeutic solutions, the approach of partner treatment to prevent reinfection is justified. However, it is apparent to clinicians and researchers that a combination of strategies is likely to be required to achieve sustained cure and support the restoration of a vaginal microbiota associated with optimal sexual and reproductive health [27, 38, 124]. This may involve the combined use for example of agents to disrupt BV biofilm [116, 117], more effective delivery of antibiotics, partner treatment, vaginal microbiome transplantation from ‘healthy’ donors with a *Lactobacillus-*dominated vaginal microbiota [125, 126], use of more promising probiotics such as Lactin-V [29], and/or agents such as lactic acid [127, 128]. For decades, we have made no impact on BV cure, and it is time to acknowledge that persisting with a one-size-fits-all approach is not only ineffective, but is unacceptable and costly to women, their relationships, and the healthcare system.

## Data Availability

Not applicable
